# Learning to Eat Vegetables in Early Life: The Role of Timing, Age and Individual Eating Traits

**DOI:** 10.1371/journal.pone.0097609

**Published:** 2014-05-30

**Authors:** Samantha J. Caton, Pam Blundell, Sara M. Ahern, Chandani Nekitsing, Annemarie Olsen, Per Møller, Helene Hausner, Eloïse Remy, Sophie Nicklaus, Claire Chabanet, Sylvie Issanchou, Marion M. Hetherington

**Affiliations:** 1 Institute of Psychological Sciences, University of Leeds, Leeds, United Kingdom; 2 School of Health and Related Research, University of Sheffield, United Kingdom; 3 Department of Food Science, University of Copenhagen, Copenhagen, Denmark; 4 CNRS, UMR6265, Centre des Sciences du Goût et de l’Alimentation, Dijon, France; 5 INRA, UMR1324, Centre des Sciences du Goût et de l’Alimentation, Dijon, France; 6 Université de Bourgogne, Centre des Sciences du Goût et de l’Alimentation, Dijon, France; INRA, France

## Abstract

Vegetable intake is generally low among children, who appear to be especially fussy during the pre-school years. Repeated exposure is known to enhance intake of a novel vegetable in early life but individual differences in response to familiarisation have emerged from recent studies. In order to understand the factors which predict different responses to repeated exposure, data from the same experiment conducted in three groups of children from three countries (n = 332) aged 4–38 m (18.9±9.9 m) were combined and modelled. During the intervention period each child was given between 5 and 10 exposures to a novel vegetable (artichoke puree) in one of three versions (basic, sweet or added energy). Intake of basic artichoke puree was measured both before and after the exposure period. Overall, younger children consumed more artichoke than older children. Four distinct patterns of eating behaviour during the exposure period were defined. Most children were “learners” (40%) who increased intake over time. 21% consumed more than 75% of what was offered each time and were labelled “plate-clearers”. 16% were considered “non-eaters” eating less than 10 g by the 5^th^ exposure and the remainder were classified as “others” (23%) since their pattern was highly variable. Age was a significant predictor of eating pattern, with older pre-school children more likely to be non-eaters. Plate-clearers had higher enjoyment of food and lower satiety responsiveness than non-eaters who scored highest on food fussiness. Children in the added energy condition showed the smallest change in intake over time, compared to those in the basic or sweetened artichoke condition. Clearly whilst repeated exposure familiarises children with a novel food, alternative strategies that focus on encouraging initial tastes of the target food might be needed for the fussier and older pre-school children.

## Introduction

Despite current recommendations and the apparent health related benefits, vegetable consumption is below the recommended level in both adults and children [1], [2]. Children dislike vegetables [3] and when given the option pre-schoolers avoid vegetables when allowed to choose lunch [4]. Children prefer foods which are high in energy density [5]–[7] and appear to accept sweet taste more than bitter taste from birth [8]. Therefore, lower energy density and bitter taste might inhibit intake of vegetables among children. Nevertheless, exposure to the taste of vegetables promotes acceptance. The mere exposure phenomenon first described by Zajonc [9] predicts that familiarisation to a stimulus results in a positive attitude to that particular stimulus. Thus, applying this to food acceptance, it is predicted that repeated experience will be effective in increasing liking and intake of novel vegetables [10]–[15].

Whilst there is extensive evidence regarding the effectiveness of repeated exposure on promoting vegetable liking and intake, whether this is equally effective across children remains unclear. Food preferences have been shown to occur through pre-natal experience and breastfeeding [16]–[18]. For example, flavours experienced in amniotic fluid or breast milk might be sufficient to promote the intake of those specific or associated flavours later in life [19], [20]. Breastfed babies are more likely to accept novel foods including vegetables compared to those who were not breastfed [20], [21], breastfeeding also affects the healthfulness of the habitual diet later in life [22], [23].

In contrast to weanlings, as children get older they become more reluctant to consume novel foods and by 2–3 years of age many develop neophobia [24]. During this stage even previously liked foods might be refused [25]. Many different techniques have been tested to promote vegetable preference and intake. Such techniques range from the relatively subtle and covert such as observational learning and social modelling [26]–[28], availability [3], hiding vegetables [29], [30], to the more overt and direct such as using social praise or tangible rewards [31]. Attempts to improve the acceptability of vegetables by offering dips and condiments [28], [32], [33] or by adding energy (flavour-nutrient learning) and/or an already liked flavour (flavour-flavour learning) yields variable results [15], [34], [35].

Repeated exposure is the simplest and most convenient method to enhance vegetable intake in children, and is ecologically valid since it mimics what mothers generally do at home when introducing new vegetables. However, mothers often give up after only 5 exposures [36] yet current recommendations suggest at least 8–10 exposures [37]. Anecdotally, mothers adopt different strategies for encouraging vegetable intake in their children [30] and these different strategies may be more or less successful depending on a general child’s temperament, prior experience with vegetables and eating traits. For example, fussy eaters are likely to refuse to try new foods whereas children who are less fussy might be more receptive to any new foods including vegetables. Measuring eating traits [38] which are stable over time [39] such as fussy eating and enjoyment of food offers a means to predict eating patterns including those children who respond well to repeated exposure and those who do not.

The current study was designed to investigate three questions; what individual characteristics predict initial acceptance of a novel vegetable, what individual characteristics predict patterns of acceptance (intake) over time, for example which characteristics predict those children who consume everything offered compared to those who do not, and what individual characteristics predict the effectiveness of repeated exposure in promoting vegetable intake. Data from three investigations using the same target vegetable products, and following the same procedure in the UK [40], Denmark [41] and France [42] were combined to examine the impact of characteristics of the child such as age, BMI, eating traits and diet history (breastfeeding duration, age of introduction of solid food) on initial vegetable acceptance, pattern of intake during repeated exposure, and effectiveness of repeated exposure on learning to like this novel vegetable. Children were also grouped into eating categories, using the pattern of their intake during the exposure period, and logistic regressions were conducted to investigate predictors of category membership.

## Materials and Methods

### Participants

Managers of private day care nurseries and parents of preschool children were invited to take part in the investigation. A total of 403 preschool children from the UK (n = 108, aged 6–36 m), France (n = 123 aged 4–8 m) and Denmark (n = 172, aged 6–36 m) were recruited for the study between Jan and May 2011. Children were enrolled if they were aged between 4 and 38 months at the beginning of the study and for the French, younger cohort they were included if the introduction of complementary foods was started more than 2 weeks and less than 2 months prior to the start of the study. Children were excluded from taking part in the investigation if they had any known food allergies.

### Ethics Statement

The studies were approved by the University of Leeds, Institute of Psychological Science ethics committee (UK), Comité de Protection de Personnes Est I Bourgogne (France) and after reviewing the study protocol the study was found not to require formal approval by the Copenhagen Regional Research ethics committee. The study procedures complied with the Helsinki Declaration. Written parental consent was given for the participating children in the three countries.

### Experimental Design and Measurements of Intake

Pre-intervention levels of intake were initially evaluated. Children were given up to 200 g (2×100 g pots) of basic artichoke puree, and their intake was weighed. For the intervention, children were randomly assigned to one of three groups: repeated exposure (basic artichoke puree, n = 112), flavour-flavour learning (basic artichoke puree with added sweetness, n = 112) or flavour-nutrient learning (basic artichoke puree with added energy n = 108). Each child received 5–10 exposures to one of the purees (variation due to unplanned absences from nursery) during a state of hunger, either before a main meal or as an afternoon snack (UK and Denmark) or at the beginning of a meal (France). In UK children were offered 100 g per exposure and in Denmark and France children were offered up to 200 g. Finally, the effectiveness of the intervention was assessed by offering 200 g of basic artichoke, and the intake was weighed. Detailed descriptions of the study have been previously published elsewhere [40]–[42]. Intake was measured before and after the intervention and throughout the exposure period. Change in intake following the intervention was calculated by subtracting the baseline from the post-intervention intake.

In order to characterise the patterns of intake for each child during the intervention period, linear regressions for each child were calculated, with weight of food consumed on each trial as the outcome variable, and exposure number as the predictor variable. This provided a value for each child of the intercept corresponding to the predicted intake on exposure one and a value of the slope corresponding to the rate of change in consumption over successive exposures that were not overly affected by behaviour on each individual day. However, simple regressions could underestimate the rate of change of consumption for children who rapidly learned to like the puree. Therefore a quadratic regression was also calculated for each child. In the case where the quadratic term was a significant negative predictor, indicating that the rate of change was decreasing overtime, we used only the first five trials to create our linear model (n = 41). We then classified the children into one of four categories, according to the following algorithm. If the slope was a significant predictor at α = 0.1 level, and greater than 2 g/exposure, a child was classified as a “learner”. If their predicted intake at exposures 1 and 5 were greater than 75 g, and the slope was greater than - 2.5 g, they were classified as “plate-clearers”. If their predicted intake at exposure 5 was less than 10 g, they were classified as non-eaters. All other children were assigned to the category ‘others’.

### Study Foods

For the investigation baby-food grade ingredients were used, in order to meet the European regulation (Directive 2006/125/CE), because the study was conducted with children younger than 3 years old. One recipe was developed for each condition. The recipe, used for the repeated exposure condition and for the pre and post-intervention measurement, was a basic artichoke puree (48 kcal/100 g). For the flavour-flavour condition the chosen unconditioned stimulus was sweetness (sucrose, 51 kcal/100 g) and for the flavour-nutrient condition, the chosen unconditioned stimulus was a higher energy density (addition of sunflower oil, 144 kcal/100 g). The ingredients selected were baby food-grade frozen artichoke heart (France Recherche & Développement FRDP, Avignon, France), water, sucrose (Vermandoise, Peronne Cedex, France), sunflower oil (Huileries de Lapalisse, Lapalisse, France) and salt. A full description of the study foods used can be obtained elsewhere [40]–[42].

### Measurements of Individual Characteristics

#### Demographic and anthropometric information

Parents and caregivers were asked to report their child’s age and sex. Height and weight were self-reported by mothers in France and Denmark based on measurements taken by a medical doctor and recorded in the health notebook and in the UK measurements were recorded the experimenters using digital scales (Seca) and a portable stadiometer (LeicesterSMSSE-0260; Seca Model 416 infantometer). Using the WHO anthropometric calculator, weights and lengths or heights were entered (http://www.who.int/childgrowth/software/en/), for children over 12 months of age, weight-for-height z-scores were calculated, and for children 12 months of age and younger, weight-for-length z-scores were calculated.

#### Early feeding practices and child eating behavior

Parents and caregivers provided information related to early feeding practices. Thus, they answered the following questions “Did you breast feed your child, if so for how long?”, and “How old was your child when you introduced formula-milk?” to determine the duration of total breastfeeding. Parents and caregivers were asked “How old was your child when they were introduced to solid foods?” to determine age of introduction of solid food. Individual eating traits of the child were reported by parents using the Child Eating Behaviour Questionnaire [38] adapted for 15 month-old infants. The CEBQ is a validated psychometric tool that measures eating behaviour styles and individual appetitive traits. In the present study, four out of seven dimensions were evaluated: food fussiness, enjoyment of food, satiety responsiveness and food responsiveness. Items were scored on a 5-point Likert scale ranging from “never” to “always”. The Danish CEBQ was completed using a 7-point scale and so this was rescaled for comparison with the other countries.

### Output Variables and Statistical Analysis

Different output variables characterising children’s eating behaviour were considered. First, the initial intake of the basic artichoke at pre-intervention was considered. Second, the change in intake of basic artichoke puree from pre- to post-intervention was calculated. Third, the intercept and slope, which characterise the pattern of intake during the exposure phase, were calculated by individual regressions.

Correlations between individual characteristics were investigated using Kendall’s tau.

Multiple linear regressions were used to identify individual characteristics which predicted the initial intake before the intervention, the change in intake from pre- to post-intervention, the predicted intake on exposure one during intervention (intercept), and the rate of change of consumption over successive exposures (slope). Z-scores for all the scalar predictor variables (age, total breastfeeding duration, BMI z-score, enjoyment of food, food fussiness, food responsiveness, satiety responsiveness and age of introduction of solid food) were calculated and entered into the model as predictors, along with experimental condition, simultaneously. The normality of the distribution of the residuals was tested with the Shapiro-Wilk test. Where this assumption was not met, robust regression was carried out using iterated re-weighted least squares. We report only the significant predictors, that is those for which the gradient co-efficient was significantly different from zero (alpha <0.05) as assessed by t-test.

One-way ANOVA was used to investigate differences in individual characteristics between eating categories (learners, non-eaters, plate-clearers and others) and Bonferroni corrected pairwise comparisons were used to interpret significant differences. Chi-square tests were used to investigate frequency of eating category in each country and frequency of eating category across experimental conditions. Logistic regressions were used to predict which variables characterised eating category. Multicollinearity was assessed using the variance inflation factor (VIF), and models with VIF greater than 10 were disregarded [43]. All data are presented as means ± SD and the alpha value was set at 0.05 except for individual regressions.

## Results

### Participant Characteristics

403 children were recruited to take part and data from 71 children were excluded due to one of the following reasons: not meeting the inclusion criteria, withdrawal from the study or insufficient exposures during the intervention period. Data for 332 pre-school children aged between 4 and 38 months (mean age 18.9±9.95 months) are presented. Table 1 displays the demographic characteristics overall and for each country.

**Table 1:** Participant characteristics of pre-school children who took part in the intervention (Means ± SD) overall and split by country (DK = Denmark, UK = United Kingdom, FR = France)*.

	All		DK		UK		FR	
	N =		N =		N =		N =	
Number of participants	332		165		72		95	
Age (months)	332	18.92 (9.95)	165	24.01 (7.03) a	72	23.56 (7.75) a	95	6.57 (0.92) b
Sex	332	M = 175 F = 157	165	M = 86 F = 79	72	M = 32 F = 40	95	M = 57 F = 38
BMI z-score	228	0.36 (1.21)	103	0.57 (1.12) b	47	1.14 (0.74) a	78	−0.39 (1.15) c
Duration of exclusive breastfeeding (weeks)	222	13.56 (8.61)	98	16.35 (6.92) a	35	14.17 (8.95) a, b	89	10.25 (9.09) b
Duration of total breastfeeding (weeks)	240	21.27 (17.94)	114	23.1 (13.07) a	35	31.09 (30.9) a	91	15.21 (14.16) b
Age of introduction of solid food (weeks)	248	21.24 (4.48)	118	20.02 (4.79) b	35	20.57 (4.16) b	95	23 (3.57) a
Enjoyment of food	247	4.11 (0.61)	119	4.09 (0.59)	35	3.94 (0.63)	93	4.21 (0.61)
Satiety responsiveness	248	2.63 (0.91)	119	3.34 (0.37) a	35	2.84 (0.47) b	94	1.65 (0.58) c
Food fussiness	247	2.48 (0.79)	119	3.02 (0.32) a	35	2.3 (0.78) b	93	1.87 (0.75) c
Food responsiveness	248	2.49 (0.74)	119	2.54 (0.75)	35	2.25 (0.63)	94	2.52 (0.77)

*not all parents answered all questions.

Means with a different letter are significantly different (a, b, c).

There were significant differences in the age of children between each country (F(2,329) = 268.7, p<0.001). This was due to differences between France and both Denmark and UK (both p<0.001). ANOVA demonstrated a main effect of country for length of time of exclusive breastfeeding (F(2,219) = 13.07, p<0.001) with differences existing between Denmark and France (p<0.001). Differences between the UK and Denmark were borderline (p = 0.051). ANOVA also demonstrated significant differences between the countries for total breastfeeding duration (F(2,237) = 12.04, p<0.001). This was due to differences between France and both Denmark (p = 0.004) and UK (p<0.001). The difference between Denmark and UK was borderline significant (p = 0.051). There were also differences between all countries in the age of introduction of solid food (F(2,245) = 13.3, p<0.001), with differences existing between France and both Denmark (p<0.001) and UK (p = 0.01). Additionally there were differences between all countries for the BMI z-score (F(2,225) = 34.23, p<0.001), satiety responsiveness (F(2,245) = 342.1, p<0.001), and food fussiness (F(2,244) = 103.0, p<0.001). There was no difference between the countries for food responsiveness or enjoyment of food.

### Correlations between Measured Individual Characteristics

Table 2 shows the pattern of correlations between the different measures of individual characteristics. The Shapiro-Wilk test of normality revealed significant deviations from normality for most variables. Therefore, Kendall’s tau was calculated to indicate associations between the variables. Age was significantly positively correlated with BMI z-score, satiety responsiveness, food fussiness, and duration of breastfeeding. Age was negatively correlated with enjoyment of food and age of introduction of solid food. BMI z-score had a positive correlation with duration of breastfeeding, satiety responsiveness and food fussiness; this last correlation is to be expected since age correlated with BMI z-score and with food fussiness. Enjoyment of food had a significant negative correlation with satiety responsiveness. Satiety responsiveness also positively correlated with food fussiness and duration of breastfeeding and had a significant negative correlation with age of introduction of solid food. Food fussiness had a significant positive correlation with duration of breastfeeding, which is to be expected given that older children had been breastfed for longer and were also more food fussy. Food fussiness also had a significant negative correlation with age of introduction of solid food.

**Table 2:** Patterns of correlations between all measures taken in pre-school children.

	BMI z-score	Enjoymentof food	Satietyresponsiveness	Foodfussiness	Foodresponsiveness	Duration of totalbreastfeeding(weeks)	Age of introductionof solid food(weeks)
Age (months)	.24 ***	−.13**	.48 ***	.42 ***	−.01	.22 ***	−.15**
BMI z-score		.03	.16 ***	.12 **	.06	.11 *	−.06
Enjoyment of food			−.11 *	.001	.06	−.02	.03
Satiety responsiveness				.45 ***	.08	.17 ***	−.24 ***
Food fussiness					.003	.13 **	−.18 ***
Food responsiveness						−.02	.05
Duration of total breastfeeding (weeks)							.06

*p<0.05, **p<0.01, ***p<0.001.

### Predictors of Pre-intervention Intake

We calculated regression models to investigate the predictors of the actual intake of the novel target vegetable in the pre-intervention phase. The only significant predictor of the amount of artichoke consumed during the pre-intervention phase was age (b = −24.25 (6.28), β = −0.40 (0.10), p<0.001, overall model adj R^2^ = 0.3, p<0.001). With robust regression, age (b = −16.8 (4.9), β = −0.28 (0.08), p<0.001) and satiety responsiveness (b = −16.1 (4.7), β = −0.26 (0.08), p<0.001) were both significant predictors, with younger and less satiety responsive children consuming more of the novel vegetable in the pre-intervention period.

### Predictors of Change in Intake from Pre- to Post-intervention

Overall the model was weak, with adj R^2^ = 0.05, p = 0.04. The predictors of change in artichoke intake were enjoyment of food (b = 12.9 (5.9), β = 0.17 (0.08), p = 0.03) and being in the flavour-nutrient learning condition (b = −35.5 (14.9), β = −0.20 (0.09), p = 0.02). With robust regression these two variables remained significant predictors; enjoyment of food (b = 13.1 (6.0), β = 0.17 (0.08), p = 0.03) and being assigned to the flavour-nutrient learning condition (b = −31.8 (15.1), β = −0.18 (0.09), p = 0.04). The change in intake from pre- to post-intervention could suffer from ceiling effects for children who ate most during both pre- and post-intervention tests. Therefore, we repeated this analysis using only children who ate less than 50 g at pre-intervention. This revealed a highly significant model (adj R^2^ = 0.21, p<0.001) with age (b = −32.56 (9.90), β = −0.43 (0.13), p = 0.001) and enjoyment of food (b = 13.88 (6.34), β = 0.21 (0.09), p = 0.03) as significant predictors of the change in intake. With robust regression age (b = −37.5 (10.1), β = −0.49 (0.13). p<0.001) and enjoyment of food (b = 14.3 (6.5), β = 0.21 (0.10), p = 0.03) were still the only significant predictors.

### Predictors of Initial Intake (Intercept) during the Intervention

The model was a good predictor of the intercept (adj R^2^ = 0.49, p<.001), with age (b = −27.5 (4.88), β = −0.48 (0.08), p<0.001), enjoyment of food (b = 6.76 (3.07), β = 0.12 (0.05), p = 0.0) and satiety responsiveness (b = −9.87 (4.88), β = −0.17 (0.08), p = 0.04) as the significant predictors. Younger children consumed more during the initial exposures than older children and children who scored higher on enjoyment of food and lower on satiety responsiveness consumed more during the initial exposures than those with lower scores on these variables.

### Predictors of Slope during the Intervention

During the intervention period the children were exposed to one of three versions of the artichoke puree. In order to investigate whether the impact of the factors in the model differed by group, linear regressions were constructed in which the interaction terms of the variables with the experimental condition were entered as predictors. For the slope model, no interaction terms were significant predictors. However, for the intercept, the interaction terms were significant for the predictors of age, enjoyment of food, and satiety responsiveness. Therefore, the individual linear regressions were conducted for each experimental condition separately to further understand the impact of individual differences within the three groups. This found that in the repeated exposure group (adj R^2^ = 0.40, p<.001), age (b = −27.12 (8.50), β = −0.51 (0.16), p = 0.002) and enjoyment of food (b = 11.80 (5.65), β = 0.22 (0.10), p = 0.04) were significant predictors of intercept. However, in the flavour-flavour learning group (adj R^2^ = 0.62, p<0.001) only age was a significant predictor (b = −46.52 (9.25), β = −0.66 (0.13), p<0.001). In the flavour-nutrient learning group (adj R^2^ = 0.48, p<0.0001), only satiety responsiveness (b = −24.53 (8.99), β = −0.40 (0.15), p = 0.009) predicted initial intake.

As there were no interactions with group for predicting slope, a simpler regression model was constructed with the experimental condition, and z-scored age, total breastfeeding duration, BMI z-score, enjoyment of food, food fussiness, food responsiveness, satiety responsiveness, and age of introduction of solid food as predictors. This produced a significant model (adj R^2^ = 0.05, p = 0.03), with experimental condition (being assigned to the flavour-nutrient learning condition) as the only significant predictor of slope (b = −4.31(2.04), β = −0.17 (0.08), p = 0.04). That is, those children in the flavour-nutrient learning condition had a flatter slope than those in either of the two other conditions. With robust regression, being in the flavour-nutrient group was the only predictor of slope (b = −4.8 (1.8), β = −0.19 (0.07), p = 0.009).

### Eating Categories (Plate-clearers, Non-eaters, Learners and Others) and Their Predictors

**Figure 1:**
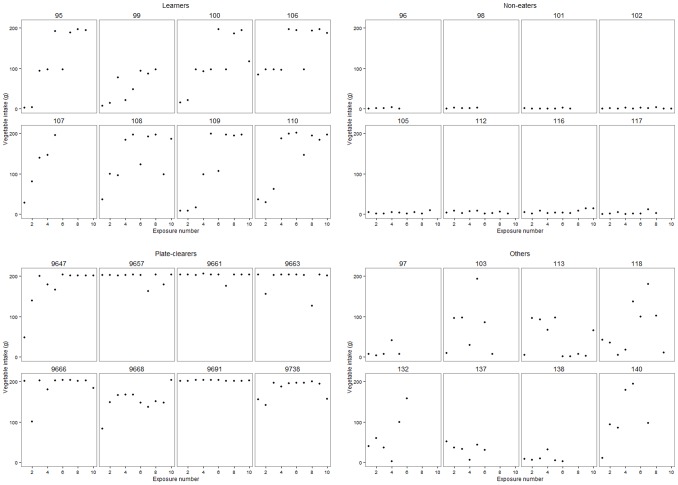
Eating categories: examples of individual profiles of intake (g) of artichoke over the intervention period. (Numbers for each case represent participant ID).

Most children, 40%, in our sample were characterised as “learners” (n = 133), 16% (n = 53) of the children were classified as “non-eaters”, 21% (n = 70) as “plate-clearers”, and 23% (n = 76) were classified as “others” since their pattern did not fit any of the other categories in a systematic way. Figure 1 shows typical intake profiles of a sample of children from each of the eating categories (with child ID presented above each profile).

Significant differences in the age of children in each eating category were observed (F(3,328) = 33.8, p<0.001). Differences were significant between all eating categories except between the learners and “others”. ANOVA also demonstrated significant differences between the eating categories for satiety responsiveness (F(3,244) = 18.22, p<0.001) this was due to plate-clearers differing from both learners (p<0.001) and others (p = 0.008), and non-eaters differing from both plate-clearers (p<0.001) and others (p = 0.008). Food fussiness also differed significantly between the categories of eaters, (F(3,243) = 19.7, p<0.001). Non-eaters were significantly more food fussy than others and plate-clearers (both p<0.001) and learners (p = 0.02). Learners were more food fussy than others (p = 0.01) and plate-clearers (p<0.001). There was no difference between the categories of BMI z-score, enjoyment of food, food responsiveness, total breastfeeding duration, or age of introduction of solid food (Table 3).

**Table 3:** Eating category individual characteristics of pre-school children (Means ± SD).

	Learners	Non-eaters	Plate-clearers	Others
N	133	53	70	76
Mean consumption during intervention (g)	94.0 (45.1) b	3.9 (2.0) d	118.3 (39.5) a	65.4 (39.5) c
Predicted initial intake (Intercept, g)	35.4 (41.5) c	3.6 (3.6) d	118.9 (36.4) a	63.0 (39.5) b
Rate of change in intake (Slope, g/exp)	17.2 (11.8) a	0.0 (0.8) b	3.0 (5.2) b	1.5 (9.5) b
Age (months)	20.0 (9.4) b	27.8 (5.7) a	12.4 (8.0) c	16.8 (9.9) b
BMI z-score	0.44 (1.23)	0.52 (0.96)	0.38 (1.32)	0.08 (1.14)
Enjoyment of food	4.1 (0.6)	3.9 (0.7)	4.2 (0.6)	4.1 (0.6)
Satiety responsiveness	2.8 (0.9) b	3.2 (0.5) a	2.0 (0.8) c	2.5 (0.9) b
Food fussiness	2.7 (0.7) b	3.1 (0.5) a	2.0 (0.8) c	2.3 (0.8) c
Food responsiveness	2.5 (0.7)	2.4 (0.9)	2.0 (0.8)	2.5 (0.8)
Duration of total breastfeeding (weeks)	20.8 (14.4)	23.0 (14.9)	19.7 (16.8)	22.7 (25.2)
Age of introduction of solid food (weeks)	20.7 (4.6)	21.0 (5.6)	21.7 (4.0)	21.8 (3.9)

Means with a different letter are significantly different (a, b, c, d).

Chi-square tests revealed significant differences between distribution of eating categories across the different countries (Chi-sq (6) = 70.8, p<0.001) (Table 4). There were more plate-clearers and fewer non-eaters in the French sample than in the other two countries. This is likely to be because the French children were significantly younger than the other children in the study. There were fewer plate-clearers in the Danish sample, more learners and non-eaters compared to the other countries.

**Table 4:** Distribution (frequency and percentage of sample) of eating categories across countries.

	France	Denmark	UK
Learners	28 (29%)	82 (50%)*	23 (32%)
Non-eaters	0 (0%)**	38 (23%)**	15 (21%)
Others	26 (27%)	35 (21%)	15 (21%)
Plate-clearers	41 (43%)**	10 (6%)**	19 (26%)

(*chi-sq p<0.05, **contributes to chi-sq p<0.01).

Those in bold represent categories where there were more pre-school children than expected, those in italic represent those categories where there were less than expected.

Chi-square tests also revealed significant differences between distribution of eating categories across experimental conditions (Chi–sq = (6) = 20.3, p = 0.002) (Table 5). Children who were in the flavour-nutrient group were more likely to be “others”, and less likely to be learners.

**Table 5:** Distribution (frequency and percentage of sample) of eating categories across conditions.

	Flavour-flavour learning	Flavour-nutrient learning	Repeated exposure
Learners	51(46%)	30 (28%)*	52 (46%)
Non-eaters	6 (14%)	23 (21%)	14 (13%)
Others	17 (15%)	37(34%)*	22 (20%)
Plate-clearers	28 (25%)	18 (17%)	24 (21%)

(*chi-sq p<0.05).

Those in bold represent categories where there were more pre-school children than expected, those in italic represent those categories where there were less than expected.

### Predictors of Eating Category

Two logistic regression models were constructed to discriminate non-eaters from learners, and non-eaters from plate-clearers. The first model was successful at discriminating learners from non-eaters (Chi-sq (10) = 20.8, p = 0.02). The only significant predictors of eating category was food fussiness (b = −1.04 (0.50), Z = −2.09, p = 0.04), although age approached significance (b = −0.72 (0.39) Z = −1.85, p = 0.06). That is, younger children were more likely to be learners than non-eaters, and children who scored higher on food fussiness were more likely to be non-eaters than learners. The second model (plate-clearers vs. non-eaters) was a highly predictive model (Chi-sq (10) = 73.8, p<0.001), with age (b = 3.90 (1.32), Z = −2.95, p<0.001) as the only significant predictor, although food fussiness approached significance (b = 2.02 (1.16), Z = −1.917, p = 0.055). Younger children were more likely to be plate-clearers than older children and children with higher food fussiness scores were more likely to be non-eaters.

## Discussion

The aim of the current study was to investigate how individual characteristics influence initial acceptance and effectiveness of repeated exposure to a novel vegetable. Our results demonstrated that the younger children were less fussy, enjoyed food more and had lower satiety responsiveness, representing a profile of characteristics that together contributed to increased acceptance of a novel food. Change in target vegetable intake (post - pre intervention) was predicted by age and enjoyment of food, with younger children and those with higher enjoyment of food scores being more responsive to repeated exposure. Age, enjoyment of food and satiety responsiveness were significant predictors of initial intake in the intervention period. Younger children consumed more as did those children who scored higher on enjoyment of food and lower on satiety responsiveness. Recipe was also important since experimental condition was the only significant predictor of the rate of change in intake over time, with those children in the flavour-nutrient learning condition (receiving a more energy-dense purée) demonstrating a flatter slope. The present investigation demonstrated that children respond differently to an intervention aimed at enhancing vegetable intake. Four categories of eaters were identified, plate-clearers, non-eaters, learners and others. Children who demonstrated a gradual increase over time were classified as learners and constituted the largest group. Age, food fussiness and being in the flavour-nutrient learning condition were predictors of eating category membership. Younger children were more likely to be learners than non-eaters and those children scoring higher on food fussiness were more likely to be non-eaters than learners. Similarly, younger children were more likely to be plate-clearers than older pre-school children.

The age of child is key when introducing novel foods [44]. Age predicted initial intake of the novel vegetable both pre-intervention and during the initial exposure of the intervention period, with younger children consuming more compared to older children. Plate-clearers were also younger and less fussy whilst older children ate less consistently, were more likely to be non-eaters and were fussier compared to younger children. Successful repeated exposure is dependent upon tasting even small amounts of the target food [45], [46]. Thus, repeated exposure is more likely to be effective at a time when most tastes are easily accepted, namely the weaning period. The first year of life presents a window of opportunity before the onset of food neophobia, which then peaks around 2–6 years [44], [47], [48], thus introducing novel foods such as different vegetables is optimal earlier rather than later. Repeated exposure has been reported to reduce neophobia [10], however, in pre-school children sufficient taste exposures are required to establish learned safety of novel foods [49]. Therefore, alternative methods that focus on encouraging initial tastes of the target food might be needed for the fussier and older pre-school children.

Learners were approximately 6 m younger than the non-eaters and this might reflect the period before neophobia develops. Learners in this sample scored higher on satiety responsiveness, and were relatively fussy compared to the plate-clearers and others. Food fussiness has been reported to correlate positively with satiety responsiveness [38], [50] and negatively with enjoyment of food and food responsiveness [38], [51]. In the current study food fussiness and satiety responsiveness, both “food avoidant” appetitive traits, positively correlated with age [50]. Age was also negatively correlated with enjoyment of food with younger children scoring higher on this scale.

Children in the “other” eating category had high initial intake, which dropped off or failed to increase over time. These children have low levels of food fussiness (same as plate-clearers) but higher levels of satiety responsiveness (same as learners). It is possible that these children were more subject to learned satiety, as there were more than the expected number of children in the flavour-nutrient condition within this group.

Children in the flavour-nutrient learning condition consumed less artichoke over the intervention period confirming previous investigations where energy was added to vegetables from carbohydrates [35] or a mixture of carbohydrates and fat [15]. It appears that flavour-nutrient learning is not effective in promoting intake of vegetables in young children. The current investigation demonstrated the benefit of repeated exposure to a novel food [10]–[14], [32], [52] and to a limited extent some advantage of flavour-flavour learning [34], [53] on promoting its acceptance. Although children prefer energy dense foods [6], [7], [54], adding oil directly to a novel vegetable changes both taste and texture and might reduce liking. However, de Wild et al. [15] demonstrated that pre-school children reported liking a high energy dense soup more than a matched low energy dense soup, but this preference did not affect intake. One possible explanation for this is expected satiation and learned satiety [55]. In support of this, satiety responsiveness was the only significant predictor of intake in the flavour-nutrient learning condition, suggesting detection of extra energy such as oil might limit intake. Alternatively children might simply have preferred the sweeter lower-fat version [34], [56], [57] or the pure, unadulterated taste of vegetables in the basic artichoke puree. Ahern et al. [58] reported that caregivers use fats such as butter in small amounts, however, little is known about how fat content and palatability might be manipulated to optimise vegetable intake in young children [56].

The findings of this study should be interpreted in light of the potential limitations. For example self-report measures were used to investigate height and weight in the Danish sample and for the French sample this was based on the most recent paediatrician’s record of height/length and weight. Similarly age of introduction of solid food, duration of breastfeeding were self-reported and these might not be completely accurate, especially in the older children. This might partially explain the inverse relationship observed between age and age of introduction of solid food. The purees were served cold to the children in the UK and Denmark and warm to the French children and this might have influenced intake. However, this is unlikely since a novel food was used and the children would not have developed any learned expectations that the food should be served warm. Therefore, it is unlikely that warming the food would have had a significant impact on the taste to enhance palatability and intake. During the exposures the Danish and French children were given access up to 200 g during the intervention, therefore increasing the possibility of these children consuming more than UK sample who were offered 100 g. However, there was no evidence that the Danish children consumed more artichoke during the intervention. Additionally, the younger cohort included in this study were exclusively French and so we were not able to include country as a predictor in the models. To eliminate the confounding effect of this and the fact that the UK children and Danish children were offered different amounts the regressions were performed without the French data (data not shown), including the factor of country as a predictor. Interestingly age remained a significant predictor of pre-intervention artichoke intake, change in artichoke intake and of the intercept. This demonstrated indirectly that the portion size offered had no impact on the age effects observed in the current study. Age was no longer significant predictor of the rate of change as indicated by the slope. All other significant predictors became non-significant. For the analysis discriminating learners from non-eaters, no predictors were found. Yet, for the test discriminating plate-clearers from non-eaters, age remained a significant predictor. It is worth noting that the removal of the French data resulted in a dramatic reduction in number of participants and potentially power. Finally, whilst a positive effect of repeated exposure was observed in the younger pre-school children, it remains to be determined how long this lasts, although durable effects were observed at three [42] and six months post intervention [41] it is not clear if the effects would be stable beyond this time.

This is the first study, to our knowledge, to investigate the role of individual differences in response to novel vegetable exposure. It is clear that children respond differently to repeated exposure. This suggests that recommendations to improve vegetable intake in children should take account of individual differences. Novel vegetables are best introduced when children are young during a period when novel foods are readily accepted and before the onset of neophobia. Food preferences are formed early on, tend to be fixed from early childhood and track into adulthood [4] it is therefore critical that a healthful diet is established early. Whilst repeated exposure might be effective for most children, for older and fussier children, other approaches are needed to improve acceptance. Alternative techniques such as the use of dips and sauces might be an effective way of encouraging these fussy eaters to try the target food [28], [32], [33]. Alternatively providing vegetables by stealth would ensure that these children are gaining the nutritional benefits of consuming vegetables [29], [30]. Offering tangible non-food rewards [31] may be more effective in these children. In future it will be of interest to compare the efficiency of these different strategies to improve vegetable intake in older and fussier pre-school children.
